# Supplementary material for: Modeling glioblastoma heterogeneity as a dynamic network of cell states
Source: Mol Syst Biol. 2021 Sep 16;17(9):e10105. doi: 10.15252/msb.202010105 (PMC8444284; doi:10.15252/msb.202010105)
Supplement: Supplementary file 5 — Source Data for Figure 3 [file MSB-17-e10105-s001.zip › Figure3A_sourcedata/GSEA_3065/hallmarks_state6.GseaPreranked.1623416308702/HALLMARK_INFLAMMATORY_RESPONSE.html]

Details for gene set HALLMARK\_INFLAMMATORY\_RESPONSE[GSEA]

|  || Dataset | state6 |
| Phenotype | NoPhenotypeAvailable |
| Upregulated in class | na\_neg |
| GeneSet | HALLMARK\_INFLAMMATORY\_RESPONSE |
| Enrichment Score (ES) | -0.4192106 |
| Normalized Enrichment Score (NES) | -1.5617434 |
| Nominal p-value | 0.010434783 |
| FDR q-value | 0.024549827 |
| FWER p-Value | 0.239 |
Table: GSEA Results Summary

  

Fig 1: Enrichment plot: HALLMARK\_INFLAMMATORY\_RESPONSE      
 Profile of the Running ES Score & Positions of GeneSet Members on the Rank Ordered List

  

| PROBE | GENE SYMBOL | GENE\_TITLE | RANK IN GENE LIST | RANK METRIC SCORE | RUNNING ES | CORE ENRICHMENT || 1 | CDKN1A |  |  | 1 | 0.477 | 0.0870 | No |
| 2 | BTG2 |  |  | 63 | 0.234 | 0.1229 | No |
| 3 | RGS16 |  |  | 239 | 0.164 | 0.1328 | No |
| 4 | EIF2AK2 |  |  | 678 | 0.108 | 0.1023 | No |
| 5 | KLF6 |  |  | 1424 | 0.067 | 0.0292 | No |
| 6 | ABI1 |  |  | 1465 | 0.066 | 0.0366 | No |
| 7 | RHOG |  |  | 1688 | 0.057 | 0.0216 | No |
| 8 | RAF1 |  |  | 1924 | 0.050 | 0.0038 | No |
| 9 | PDE4B |  |  | 2004 | 0.048 | 0.0035 | No |
| 10 | MYC |  |  | 2081 | 0.045 | 0.0031 | No |
| 11 | ACVR2A |  |  | 2368 | 0.037 | -0.0229 | No |
| 12 | IRF1 |  |  | 2377 | 0.037 | -0.0171 | No |
| 13 | NMI |  |  | 2414 | 0.036 | -0.0147 | No |
| 14 | AHR |  |  | 2711 | 0.029 | -0.0434 | No |
| 15 | P2RX7 |  |  | 2929 | 0.023 | -0.0639 | No |
| 16 | SLC31A1 |  |  | 2963 | 0.023 | -0.0636 | No |
| 17 | RELA |  |  | 2971 | 0.023 | -0.0603 | No |
| 18 | ABCA1 |  |  | 3161 | 0.019 | -0.0785 | No |
| 19 | AXL |  |  | 3293 | 0.016 | -0.0907 | No |
| 20 | IRF7 |  |  | 3602 | 0.009 | -0.1243 | No |
| 21 | SERPINE1 |  |  | 3811 | 0.004 | -0.1473 | No |
| 22 | HIF1A |  |  | 3882 | 0.003 | -0.1548 | No |
| 23 | PLAUR |  |  | 3956 | 0.001 | -0.1629 | No |
| 24 | NDP |  |  | 4018 | 0.000 | -0.1698 | No |
| 25 | KCNJ2 |  |  | 4147 | -0.002 | -0.1841 | No |
| 26 | GABBR1 |  |  | 4201 | -0.003 | -0.1896 | No |
| 27 | KIF1B |  |  | 4348 | -0.006 | -0.2052 | No |
| 28 | CSF1 |  |  | 4350 | -0.006 | -0.2043 | No |
| 29 | TAPBP |  |  | 4513 | -0.009 | -0.2212 | No |
| 30 | CHST2 |  |  | 4821 | -0.015 | -0.2537 | No |
| 31 | NFKB1 |  |  | 4949 | -0.017 | -0.2651 | No |
| 32 | CX3CL1 |  |  | 5266 | -0.024 | -0.2970 | No |
| 33 | SCN1B |  |  | 5399 | -0.026 | -0.3073 | No |
| 34 | PSEN1 |  |  | 5429 | -0.027 | -0.3057 | No |
| 35 | PTPRE |  |  | 5684 | -0.032 | -0.3289 | No |
| 36 | SRI |  |  | 6182 | -0.042 | -0.3781 | No |
| 37 | RIPK2 |  |  | 6203 | -0.043 | -0.3726 | No |
| 38 | MET |  |  | 6611 | -0.053 | -0.4095 | Yes |
| 39 | ITGA5 |  |  | 6637 | -0.054 | -0.4025 | Yes |
| 40 | CD82 |  |  | 6649 | -0.054 | -0.3939 | Yes |
| 41 | OSMR |  |  | 6835 | -0.060 | -0.4042 | Yes |
| 42 | HRH1 |  |  | 6947 | -0.063 | -0.4054 | Yes |
| 43 | IFNGR2 |  |  | 6997 | -0.065 | -0.3991 | Yes |
| 44 | NFKBIA |  |  | 7027 | -0.066 | -0.3905 | Yes |
| 45 | ATP2C1 |  |  | 7132 | -0.069 | -0.3898 | Yes |
| 46 | NAMPT |  |  | 7188 | -0.071 | -0.3832 | Yes |
| 47 | CALCRL |  |  | 7419 | -0.078 | -0.3952 | Yes |
| 48 | ADORA2B |  |  | 7469 | -0.080 | -0.3862 | Yes |
| 49 | SLC7A1 |  |  | 7535 | -0.083 | -0.3785 | Yes |
| 50 | HAS2 |  |  | 7560 | -0.084 | -0.3658 | Yes |
| 51 | LDLR |  |  | 7648 | -0.088 | -0.3597 | Yes |
| 52 | GNAI3 |  |  | 7760 | -0.093 | -0.3553 | Yes |
| 53 | ADRM1 |  |  | 7955 | -0.104 | -0.3585 | Yes |
| 54 | LY6E |  |  | 8146 | -0.117 | -0.3588 | Yes |
| 55 | SLC11A2 |  |  | 8160 | -0.118 | -0.3388 | Yes |
| 56 | ITGB8 |  |  | 8195 | -0.121 | -0.3205 | Yes |
| 57 | SELENOS |  |  | 8258 | -0.127 | -0.3044 | Yes |
| 58 | TPBG |  |  | 8401 | -0.146 | -0.2939 | Yes |
| 59 | BST2 |  |  | 8410 | -0.147 | -0.2679 | Yes |
| 60 | TNFAIP6 |  |  | 8434 | -0.150 | -0.2431 | Yes |
| 61 | ATP2B1 |  |  | 8436 | -0.150 | -0.2157 | Yes |
| 62 | IFNAR1 |  |  | 8460 | -0.154 | -0.1902 | Yes |
| 63 | PDPN |  |  | 8486 | -0.158 | -0.1642 | Yes |
| 64 | SLC4A4 |  |  | 8538 | -0.170 | -0.1390 | Yes |
| 65 | ATP2A2 |  |  | 8624 | -0.200 | -0.1121 | Yes |
| 66 | EMP3 |  |  | 8630 | -0.203 | -0.0755 | Yes |
| 67 | DCBLD2 |  |  | 8676 | -0.226 | -0.0393 | Yes |
| 68 | TIMP1 |  |  | 8751 | -0.290 | 0.0052 | Yes |
Table: GSEA details [plain text format]

  

Fig 2: HALLMARK\_INFLAMMATORY\_RESPONSE: Random ES distribution      
 Gene set null distribution of ES for **HALLMARK\_INFLAMMATORY\_RESPONSE**
